# Synergism between Extracts of *Garcinia mangostana* Pericarp and *Curcuma* in Ameliorating Altered Brain Neurotransmitters, Systemic Inflammation, and Leptin Levels in High-Fat Diet-Induced Obesity in Male Wistar Albino Rats

**DOI:** 10.3390/nu14214630

**Published:** 2022-11-03

**Authors:** Ranyah Shaker M. Labban, Hanan A. Alfawaz, Musarat Amina, Ramesa Shafi Bhat, Wail M. Hassan, Afaf El-Ansary

**Affiliations:** 1 Department of Food Science and Nutrition, College of Food and Agriculture Sciences, King Saud University, Riyadh 11495, Saudi Arabia; 2 Deputyship for Therapeutic Services, General, Administration of Nutrition, Ministry of Health, Riyadh 11595, Saudi Arabia; 3 Department of Pharmacognosy, Pharmacy College, King Saud University, Riyadh 11495, Saudi Arabia; 4 Biochemistry Department, College of Science, King Saud University, Riyadh 11495, Saudi Arabia; 5 Department of Biomedical Sciences, School of Medicine, University of Missouri Kansas City, Kansas City, MO 64108, USA; 6 Central Laboratory, Female Centre for Scientific and Medical Studies, King Saud University, Riyadh 11495, Saudi Arabia

**Keywords:** obesity, anti-inflammatory, mangosteen, neurotransmitters, *Curcuma*

## Abstract

This study aims to explore the effects of *Garcinia mangostana* (mangosteen) and *Curcuma longa* independently and synergistically in modulating induced inflammation and impaired brain neurotransmitters commonly observed in high-fat diet-induced obesity in rodent models. Male albino Wistar rats were divided into four experimental groups. Group I, control, obese, fed on a high-fat diet (HFD), and Group II-IV, fed on HFD then given mangosteen extract (400 mg/kg/day) and/or *Curcuma* (80 mg/kg/day), or a mixture of both for 6 weeks. Plasma pro-inflammatory cytokines, leptin, and brain serotonin, dopamine, and glutamate were measured in the five studied groups. *G. mangostana* and *Curcuma longa* extracts demonstrate antioxidant and DPPH radical scavenging activities. Both induced a significant reduction in the weight gained, concomitant with a non-significant decrease in the BMI (from 0.86 to 0.81 g/cm^2^). *Curcuma* either alone or in combination with MPE was more effective. Both extracts demonstrated anti-inflammatory effects and induced a significant reduction in levels of both IL-6 and IL-12. The lowest leptin level was achieved in the synergistically treated group, compared to independent treatments. Brain dopamine was the most affected variable, with significantly lower levels recorded in the *Curcuma* and synergistically treated groups than in the control group. Glutamate and serotonin levels were not affected significantly. The present study demonstrated that mangosteen pericarp extract (MPE) and *Curcuma* were independently and in combination effective in treating obesity-induced inflammation and demonstrating neuroprotective properties.

## 1. Introduction

Obesity is rapidly becoming one of the most important medical and public health problems of our time. Obesity is associated with a high rate of morbidity and early mortality if left untreated. Its role as a health hazard in adults has been well recognized for some time [1]. In 2018, the WHO reported that 39% of adults around the world are overweight with a body mass index (BMI) of (≥25 kg/m^2^) while 13% are obese (BMI ≥ 30 kg/m^2^) [2]. Being overweight and obese are major risk factors for several chronic diseases, including diabetes, cardiovascular diseases, and cancer. However, being overweight, obesity and associated diseases are preventable [3].

Obesity can adversely affect many organs and increases the risk of chronic diseases [4]. Obesity is considered an independent risk factor for cardiovascular diseases (CVD), type diabetes 2, dyslipidemia, and Metabolic Syndrome (MS). Other indirect effects of obesity are mediated by risk factors such as hypertension, IR, hyperglycemia, and dyslipidemia [5]. Excessive consumption of HFDs has undoubtedly contributed to the obesity epidemic. However, the mechanisms responsible for this relationship are likely to be more complex than the simple concept of energy balance.

Chronic inflammation as a major risk of obesity is characterized by high circulating levels of pro-inflammatory markers such as C-reactive protein (CRP), interferon (IFN), transforming growth factor-β (TGF-β), tumor necrosis factor-α (TNF-α) and its soluble receptors and serum amyloid A, and interleukin-1 (IL-1), IL-6, IL-8, IL-13 [6]. These elevated pro-inflammatory molecules additionally recruit more immune cells such as monocytes, neutrophils, eosinophils, mast cells, and blood platelets to yield more pro-inflammatory molecules such as nitric oxide (NO), reactive oxygen species (ROS), that is directly related to the loss of structure, function and integrity of lipids, proteins and nucleic acids, leading to different chronic diseases.

Steroidal and non-steroidal drugs are commonly used to treat inflammation. Nevertheless, these remedies have side effects such as nausea and vomiting sometimes lead to organ dysfunction [7]. This encourages many researchers to search for new anti-inflammatory compounds of plant origin, which would be safe enough and potent. Many plant extracts demonstrate a synergistic effect with each other or with modern drugs.

Plant-derived phytochemicals must be bioavailable to exert their beneficial effects. After being released from the plant and becoming bioaccessible, phytochemicals can be absorbed in the gastrointestinal tract. The absorption of these compounds can be influenced by multiple factors including solubility, interaction with other dietary ingredients, molecular transformations, different cellular transporters, metabolism, and the interaction with the gut microbiota, resulting in changes to their bioavailability [8].

*G. mangostana* L. (mangosteen) is a widely cultivated fruit tree in Southeast Asia such as Malaysia, Thailand, and Indonesia [9]. The mangosteen pericarp (MP) is used as a traditional medicine to treat multiple diseases because of its high content of several compounds identified as xanthones [10]. Experimental studies have revealed that xanthones are the major bioactive constituents, demonstrating antioxidant [11] antitumor [9], and antimicrobial activities [12]. In addition, α-mangostin compounds have anti-inflammatory activity by inhibiting the production of nitric oxide, TNF-α, and IL-8 secretion [13].

*Curcuma* is a polyphenol compound found in the spice turmeric that is used in traditional medicine [14,15,16]. It displays numerous physiological actions such as anti-inflammatory, antioxidant, and anticancer potencies. [15,16,17]. *Curcuma* blocks signaling pathways, such as nuclear factor kappa-B (NF-kappaB) and myeloid differentiation protein 2-Toll-like receptor 4 co-receptor pathways, activates peroxisome proliferator-activated receptor-gamma (PPAR-gamma) and inhibits the manufacture of pro-inflammatory cytokines, such as TNF-α and interleukin (IL)-1β.

Sears [18] introduced the theory of “an anti-inflammatory diet” to combat obesity and obesity-induced complications. These healthy/anti-inflammatory diets comprise high consumption of fruits and vegetables that are rich in phytochemicals and bioactive constituents from plants. These phytochemicals may contribute to the beneficial properties of these healthy diets due to the attenuation of chronic inflammation and thus prevent different chronic diseases [18,19].

Though, the clarification of the mechanism of these anti-inflammatory diets remains uncertain due to multiple reasons, first is that food may contain hundreds of phytochemicals [20], and our diets usually have multiple foods; second is the complexity of the digestion, absorption, and metabolism of phytochemicals; and finally the inconsistency between the high dosage demands of most phytochemicals and their low bioavailability after consuming appropriate foods/supplements in humans [8]. The interactions between phytochemicals during intestinal absorption could greatly affect the bioavailability of the bioactive compounds, which in turn affects the intensity of their bioactivities [8].

Numerous clinical and experimental studies indicate that altered brain neurotransmitters are connected to differences in feeding behavior, motivation to eat, energy expenditure, and reward learning. Based on these as well as other observations, it can be hypothesized that changes in feeding behavior in obese individuals could be a result of alterations in the brain’s serotonin and dopamine systems [21,22].

The present study aims to test the potency of MPE and *Curcuma* either independently or in combination in reducing inflammation, ameliorating leptin levels, or impaired brain neurotransmitters in HFD—an induced obese rodent model.

## 2. Materials and Methods

### 2.1. Plant Materials

Fresh fruits of *G. mangostana* (mangosteen) and rhizomes of *Curcuma longa* were purchased from local hypermarkets in Riyadh, Saudi Arabia in February 2019 and October 2019, respectively. The taxonomic identification of plant species was confirmed by Dr. Mohamed Yousef in the College of Pharmacy, King Saud University. The voucher specimen (GA-6-2019) and (CL-7-2019) have been deposited in the herbarium of the same department. The collected plant sample was freed from foreign material and washed thoroughly 2–3 times with double-distilled water and dried. The mangosteen fruits were peeled followed by the manual collection of pericarp and finally dried at ambient temperature for 10 days. Whereas, *Curcuma* rhizomes were peeled and dried in the oven at 50 °C for 3–4 days. After drying, the biomaterials were individually weighed, pulverized into a coarse powder using a commercial grinder, and stored in dark boxes at −80 °C in Freezer until further use. All local, national or international guidelines and legislation were adhered to in the production of this study.

### 2.2. Animals and Housing Conditions

All experimental procedures were carried out in the Experimental Surgery and Animal Laboratory Prince Naif Health Research Center (PNHRC) approved by the Ethical Committee of Scientific Research at KSU all animals were hosted in polypropylene cages in an environmentally controlled clean air room, with a temperature of (25 °C ± 1 °C) a 12 h light/12 h dark cycle and relative humidity of 50 ± 5%.

Twenty male albino Wistar rats, weighing 100 ± 20 g at four weeks of age, the animals were having free access to HFD to induce obesity for extra four consecutive weeks. The diet was prepared in collaboration with (PNHRC), Medicine college in King Khalid Hospital (KKH), (KSU), HFD (45% kcal from fat) was prepared by making the composition of the British company Test Diet replacing the pig fat in the composition of the company with 50% hydrogenated fats (Butter oil, Palm oil) and 50% coconut oil due as pork fat is forbidden according to the Saudi Food and Drug Regulations SFD.

The animals were fed on the HFD according to Philip and Reeves [23], High-Fat Diet was prepared according to Aslani et al. [24]. All experiments were performed in accordance with the guidelines of the National Institutes of Health for the Care and Use of Laboratory Animals and approved by the Animal Ethics Committee of King Saud University (reference no: KSU-SE-18-17). Our study was carried out in compliance with the ARRIVE guidelines.

### 2.3. Preparation of Extracts

Mangosteen pericarp and *Curcuma* rhizomes were individually subjected to cold percolation. 500 g of each powdered plant material was soaked in 1.5 L of absolute methanol for three successive days with continuous stirring at room temperature. The supernatant was through Whatman No. 1 filter paper followed by centrifugation to remove the suspended material. The residues were subjected to second and third extraction under similar conditions. Each day the dissolved portion was filtered and stored in a glass bottle. After the third extraction, all three organic filtrates were pooled and concentrated in vacuo using a rota-evaporator at ±50 °C under reduced pressure to afford a crude white (21.23 g) and dark yellow viscus (23.12 g) residues for MP and *Curcuma* rhizomes respectively. Both the extracts were stored in glass vials at −80 °C in a refrigerator prior to use.

### 2.4. Phytochemical Screening of the Plant Extracts

Qualitative analysis of plant extracts was conducted to identify the types of phytochemicals including phenolic, flavonoids, tannins, steroids, alkaloids, and saponins by obeying earlier described methods [25].

### 2.5. Quantitation of Total Phenolic Content (TPC)

The Folin-Ciocalteu procedure was applied to determine the total phenolic content of the extracts of *G. mangostana* and *C. longa* as described earlier [25]. Briefly, 0.5 mL of each test sample was individually added to a mixture of 7.5% sodium carbonate (2.5 mL) and 10% Folin-Ciocalteu (2.5 mL) reagent and kept in the dark at 25 °C for 20 min. After incubation, the absorbance of the reaction mixture was measured at 760 nm by a spectrophotometer. Gallic acid was used to obtain the standard curve and total phenolic content was calculated by the extrapolation of this curve.

### 2.6. Quantitation of Total Flavonoid Content (TFC)

The total flavonoid content of *G. mangostana* and *C. longa* extracts was determined by applying an aluminum chloride colorimetric method [26]. To a mixture of 10% AlCl_3_ (0.2 mL), 1M potassium acetate (0.2 mL), methanol (3.0 mL), and distilled water (5.6 mL), 1.0 mL of the test sample was added and incubated for 30 min at room temperature. The absorbance of the reaction mixture was measured at 420 nm by a spectrophotometer. Catechin was used as the reference standard and the total flavonoid content was calculated from the extrapolation of the standard curve.

### 2.7. Antioxidant Activity

Antioxidant activities were measured in vitro as reducing power, DPPH radical scavenging, and hydroxyl radical scavenging activity assays.

a. Reducing power assay

The reducing capability of *G. mangostana* and *C. longa* extracts was determined by following the Oyazal et al. (1986) method with slight modification [27]. Briefly, equal volumes of 0.2 M potassium buffer (2.5 mL) and 1% potassium ferricyanide (2.5 mL) were mixed. Afterward, 1.0 mL of different concentrations (5–80 μg mL^−1^) of each extract was individually added to the reaction mixture and incubated at 50 °C for 20 min. The reaction mixture was then treated with 2.5 mL of 10% TCA solution and centrifuged at 2500 rpm for 10 min. Finally, 2.5 mL of the prepared solution was mixed with equal volume ultrapure water (2.5 mL) and 0.5 mL of 0.1% ferric chloride solution. The absorbance of the reaction mixture was measured at 700 nm and the reference standard used for comparison was catechin.

b. DPPH radical scavenging assay

The ability of extracts of *G. mangostana* and *C. longa* to scavenge DPPH radical was assessed by obeying the modified method of Choi et al. [28]. Different concentrations (6.25–100 μg mL^−1^) of plant extracts dissolved in methanol were used to react with 0.135 mM DPPH solution and left undisturbed in the dark for 30 min. Both the extracts were scanned for absorbance at 517 nm after 30 min. Catechin was used as a reference compound for the comparison. The percentage of DPPH• radical inhibition was calculated by applying the following equation:

$$\mathrm{Percentage}\;\mathrm{of}\;\mathrm{inhibition}=(\frac{A_{C-}A_{s}}{A_{C}})\;\times \;100$$

where *A_c_* and *A_s_* are the absorbance of control and sample (extract or reference compound). The IC_50_ values were calculated by plotting the inhibition percentage against the compound concentration.

c. Determination of hydroxyl radical scavenging activity

The ability of the methanol extract of *G. mangostana and C. longa* to scavenge hydroxyl radicals was measured by obeying Kunchandy and Rao method with little modification [29]. 1 mL of the test sample (6.25–100 μg mL^−1^ concentration) of the test sample was added to the reaction mixture containing 20 mM phosphate buffer (pH 7.4), 2.8 mM 2-deoxy-2-ribose, 100 μM EDTA, 100 μM FeCl_3_, 100 μM ascorbic acid, and 1 mM H_2_O_2_ and incubated at 37 °C for 1 h. After 1 h of incubation, 0.5 mL of the reaction mixture was reacted with 1 mL of 1% of TBA and 1 mL of 2.8% of TCA and heated at 90 °C for 15 min. After cooling, the absorbance wavelength of the reaction mixture was scanned at 532 nm against the blank solution. The reference compound used was catechin for the comparison. The hydroxyl radical scavenging percentage was measured in a similar way as in the DPPH• radical scavenging assay.

### 2.8. Experimental Work

Twenty male Wistar albino rats weighing 100 ± 20 g; at the age of 4 weeks, Rats were housed individually in stainless steel cages under controlled environmental conditions of 25 °C, 12 h day/night cycle, the humidity of 50 ± 5%. Male rats were selected to avoid the role of ovarian hormones in weight gain. Rats were divided randomly into 4 groups, (5 rats in each group in a cage) as follows:

Group I obesity-positive control rats received HFD for 4 weeks before starting the experiment.

Group II obesity-positive rats HFD for 4 weeks before starting the experiment, and treated with a single dose of 400 mg/kg/day of MPE for 6 weeks (i.e., Stock solution of 400 mg MPE was dissolved in 5 mL water, and then 0.5 mL was orally administered/100 g body weight rat).

Group III obesity-positive rats received HFD for 4 weeks before starting the experiment, then treated with a single dose of 80 mg/kg/day of *Curcuma* for 6 weeks (i.e., Stock solution of 80 mg *Curcuma* was dissolved in 5 mL water, and then 0.5 mL was orally administered/100 g body weight rat).

Group IV obese animals received HFD for 4 weeks before starting the experiment then treated with 400 mg of mangosteen [30] and 80 mg/kg/day water extract of Curcum [31] for 6 weeks.

### 2.9. Measurement of the BMI

Body length (nose–anus length) was determined for all anesthetized rats. The body weight and body length were used to determine the BMI using the following equation. Body mass index (BMI) = Body weight (g)/body length^2^ (cm^2^). Animals were classified as obese because their BMI was higher than 0.75 (BMI = g/cm^2^) [32]. Body weight was measured weekly until the experimental period ended, and each animal was placed in a metabolic cage.

### 2.10. Collection of Samples

At the end of the experiment, animals were anesthetized with 5.0% of sevoflurane and 100% oxygen, the flow rate of sevoflurane was determined as the following formula: Flow rate (mL/min) = 0.5 × body weight (g). Blood and brain tissue samples were collected from each animal. Feces were also collected after two weeks’ start the animal model (after starting feeding with HFD and ND before treatment) and four, eight, and ten weeks (continued feeding with HFD and during treatment with MPE and *Curcuma*) from each animal.

### 2.11. Brain Tissue

After Anesthesia and Euthanasia, the whole brain was collected and washed with ice-cold phosphate-buffered saline PBS (0.02 mol/L, pH 7.0–7.2) to remove excess blood. The whole brain was divided into small pieces to help the easiness of homogenization and stored in the Eppendorf at −20 °C or −80 °C for further analysis. The whole dissected brain tissue was homogenized in 10 mL double distilled water for 2–3 min. The homogenates were then centrifuged for 10 min at 3000 rpm and 4 °C, and the supernatants were used to measure neurotransmitters.

### 2.12. Blood

At the end of the experiment and before euthanasia, the blood sample was collected from the heart of each animal using a hematocrit capillary 75 mm/75 uL. Serum was collected by centrifuging blood at 10,000× *g* at 4 °C for 10 min by using Sigma Model-3-30k & Sec: BL Made in Germany. Collected serum was stored at −80 °C for biochemical assay. All the biochemical measurements were done in the Central Research Laboratory in KSU female Campus.

### 2.13. Biochemical Assays

All the biochemical analyses were performed in duplicates.

1. Determination of brain dopamine:

Dopamine was measured in brain homogenates using a competitive ELISA kit, the product of MyBioSource. Cat. No: MBS725908. The assay was done according to the instructions provided with the kits. The sensitivity is 1.0 ng/mL.

2. Determination of brain glutamate:

Glutamate was measured in brain homogenates using a competitive ELISA kit, the product of MyBioSource. Cat. No: MBS756400. The assay was done according to the instructions provided with the kits. The sensitivity is 1.0 ng/mL.

3. Determination of brain Serotonin:

Serotonin was measured in brain homogenates using a quantitative Sandwich ELISA kit product of MyBioSource Cat. No: MBS9362408. The assay was done according to the instructions provided with the kits. The sensitivity is 1.0 ng/mL.

4. Determination of serum interleukin 6:

Interleukin-6 was measured in the serum using an ELISA kit, the product of MyBioSource Cat. No: MBS726707. The assay was done according to the instructions provided with the kits. The sensitivity is 1.0 pg/mL.

5. Determination of serum interleukin 12:

Interleukin-12 was measured in the serum using an ELISA kit, the product of MyBioSource Cat. No: MBS721942. The assay was done according to the instructions provided with the kits. The sensitivity is 1.0 pg/mL.

6. Determination of serum leptin:

Leptin was measured in the serum using an ELISA kit, the product of MyBioSource. Cat. No: MBS727499. The sensitivity is 1.0 ng/mL. All the assays were done according to the instructions provided with the kits.

### 2.14. Statistical Analysis

Quantitative measurements of cytokines (IL-6 and IL-12), leptin, and neurotransmitters are expressed as mean ± SEM. Statistical significance was determined using a one-way ANOVA with a Tukey post-hoc test; both were performed using GraphPad Prism version 6 for Windows (GraphPad Software, San Diego, CA, USA, www.graphpad.com, accessed on 6 May 2021). Significance was assigned at the level of *p* < 0.05. The receiver operating characteristics curve (ROC) analysis with the area under the curve (AUC), cutoff values, and the degrees of sensitivity and specificity were calculated. A stepwise multiple regression model was performed using IBM SPSS version 24 (IBM Corp., Armonk, NY, USA).

## 3. Results

### 3.1. Phytochemical Analysis

A preliminary phytochemical screening conducted on the methanolic extract of mangosteen pericarp and *Curcuma* rhizomes showed the presence of a variety of phytoconstituents including tannins, phenolics, flavonoids, xanthones, phytosterols, alkaloids, and saponins. Qualitative scrutiny of the extracts revealed that both extracts contained substantial content of phenolics and flavonoids. The amount of flavonoids content was higher in the *Curcuma* rhizome extract, whereas MPE was richer in phenolic compounds, particularly xanthones (Table 1).

Assays for the estimation of the total phenolic and flavonoid content of the extractives revealed that mangosteen extract contained the highest phenolic content (86.23 ± 0.62 mg GAE/g dried extract) as compared to *Curcuma* extract (72.02 ± 0.22 mg GAE/g dried extract) (Table 2). Whereas *Curcuma* extract showed higher content of flavonoids (174.24 ± 0.20 mg CE/g dried extract) in contrast to mangosteen extract (137.24 ± 1.05 mg CE/g dried extract).

### 3.2. Antioxidant Activity

The antioxidant potential of the extractives of *G. mangostana* and *C. longa* was measured by applying three in vitro models such as reducing power, DPPH radical scavenging, and hydroxyl free radical scavenging.

The reducing ability of *G. mangostana* and *C. longa* extract was assessed by reducing power assay. The result showed that both extracts were found to have a reducing effect and the effect was enhanced with the increase in the concentration of the extract. The absorbance obtained for *G. mangostana* extract and *C. longa* extract were 2.123 ± 0.05 and 2.542 ± 0.03 respectively, at 80 μg mL^−1^, indicating *G. mangostana* extract was more active. Notably, the *G. mangostana* extract exhibited higher activity than that of the reference antioxidant catechin (Table 2).

DPPH is a widely used stable free radical for evaluation of the scavenging capability of the antioxidant. The DPPH scavenging potential of methanolic extracts of *G. mangostana* and *C. longa* increased with the gradual increase in concentration. *C. longa* extract showed stronger radical scavenging which exhibited an IC_50_ of 7.48 µg mL^−1^ and was 3.03 fold lower than that of catechin (4.45 µg mL^−1^). However, *G. mangostana* extract with the IC_50_ value of 10.35 µg mL^−1^ expressed relatively lower radical scavenging activity (*p* < 0.05) (Table 2).

The hydroxyl radical is considered as most harmful radical among the radicals produced in the biological system. Fenton reaction in vitro generates the hydroxyl radicals and the capacity of the extractives to scavenge the radicals was measured (Table 2). The *C. longa* extract displayed stronger scavenging activity compared to *G. mangostana* extract with IC_50_ values of 12.37 µg mL^−1^ and 24.26 µg mL^−1^ respectively. It was observed that *C. longa* extract possesses higher scavenging potential than that of catechin (IC_50_ = 14.67 µg mL^−1^) standard antioxidant.

Data are presented as Mean ± S.D. of all the measured variables. Table 3 demonstrates the effects of mangosteen and *Curcuma* either alone or in combination on BW, weight gain, and BMI of obese rats. It is clear that both extracts induce a significant reduction in the gained weight concomitant with a non-significant decrease in the BMI (from 0.86 to 0.81 g/cm^2^). *Curcuma* either alone or in combination was more effective (BMI of 0.78 and 0.79 g/cm^2^ respectively).

**Figure 1:**
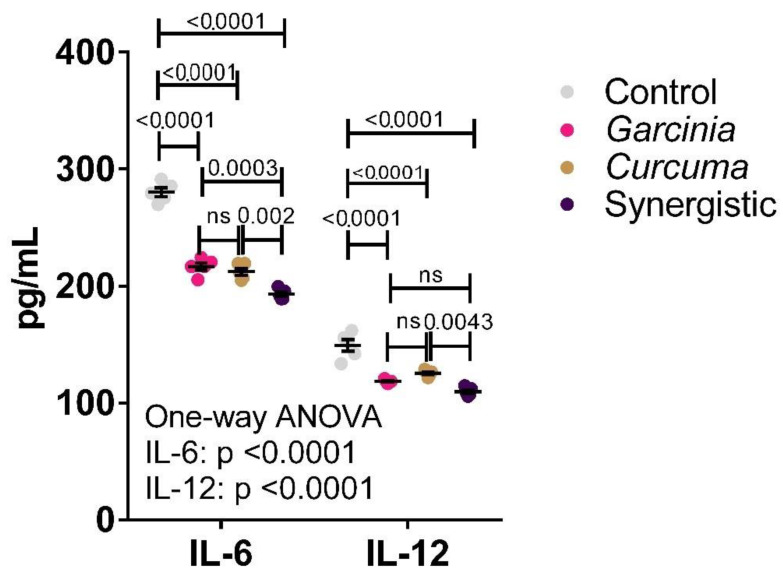
Independent and synergistic effects of *C. longa* and *G. mangostata* extracts on serum IL-6 and IL-12 in lean and HFT-induced obese rats. The mean and SEM are indicated in black. Significance was determined using a one-way ANOVA with a Tukey post-hoc test. ns: not significant.

Figure 1 presents the level of serum IL-6 and IL-12 as pro-inflammatory markers. Both extracts have demonstrated anti-inflammatory effects by inducing a highly significant reduction in the levels of these inflammatory cytokines. A synergistic effect resulting in further cytokine reduction was observed when both extracts were used in combination.

**Figure 2:**
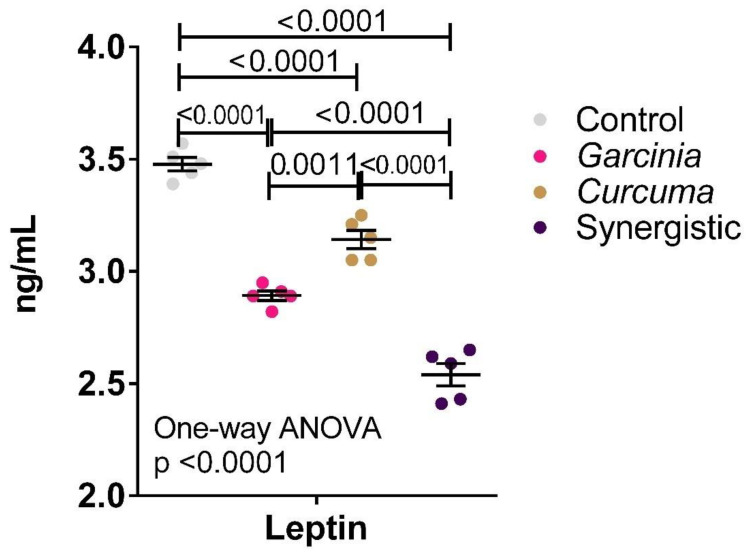
Effect of the independent and synergistic effects of *Curcuma* and Mangosteen extract on serum leptin in lean and HFT-induced obese rats. The mean and SEM are indicated in black. Significance was determined using a one-way ANOVA with a Tukey post-hoc test. ns: not significant.

Figure 2 demonstrates the anti-atherogenic effects of MPE and *Curcuma* either independently or in combination. Much lower serum leptin was observed in the three treated groups with the lowest leptin level reported in the synergistically treated group.

**Figure 3:**
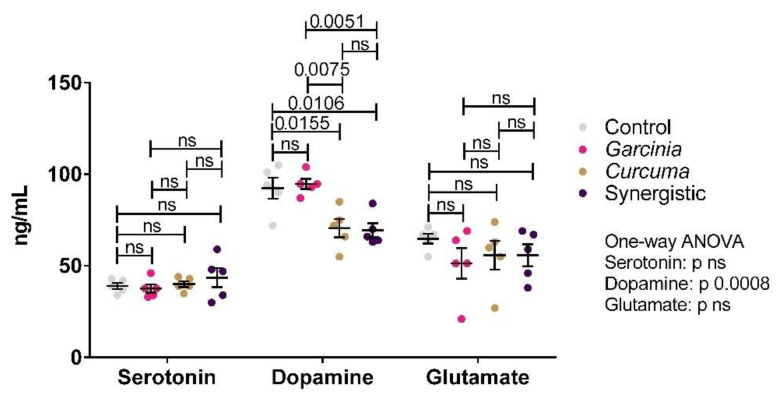
Effect of the independent and synergistic effects of *Curcuma* and Mangosteen extracts on brain neurotransmitters in lean and HFT-induced obese rats. The mean and SEM are indicated in black. Significance was determined using a one-way ANOVA with a Tukey post-hoc test. ns: not significant.

Figure 3 demonstrates the levels of serotonin, dopamine, and glutamate in brain homogenates. Significant changes were only observed in dopamine, whose levels were lowered by *Curcuma* extract treatment. MPE did not appear to have an effect.

Table 4 presents the multiple regression analysis using IL-6 and leptin as dependent variables. It can be easily noticed that 97.4% of the change in IL-6 is due to the variations in IL-12, dopamine, and leptin as markers of inflammation, brain neurochemistry, and obesity respectively. Moreover, 98.2% of the change in leptin is due to the changes in IL-6, IL-12, serotonin, and BW as markers of inflammation, brain chemistry, and obesity respectively.

Table 5 demonstrates the ROC analysis of all the measured variables in the three treated groups in relation to the control obese group. ROC-AUCs, cutoff values, specificity, and sensitivity are shown in the table.

## 4. Discussion

It is well accepted that the combined biological effects of phytochemical mixtures derived from fruits and vegetables could demonstrate different anti-oxidative, anti-inflammatory, and anti-atherogenic activities. The bioavailability impairment or enhancement caused by the co-consumption of dietary phytochemicals is of critical importance [33].

This study demonstrates that both mangosteen and *Curcuma* contain a substantial amount of phytocompounds that could benefit human health as anti-obesity, neuroprotective, antioxidant, free radical scavenging, and anti-inflammatory properties. The obtained data show that MPE contains much higher xanthones than *Curcuma* (Table 1). From the economical point of view, the dark purple MP, which is commonly treated as cultivated waste, proved as a source of xanthone, a plant secondary metabolite with several health-stimulating effects. This can find support in multiple studies which reported that α-mangostin is the major type of xanthones that is present in MPE, followed by low amounts of other xanthone derivatives, such as garcinone, gartanin, mangostanol, and mangostanin [34,35,36,37]. Both *Curcuma* and MPE contain comparable phytochemical components with MPE being richer in TPC and xanthones, while *Curcuma* has much higher TFC (Table 2). Both recorded significantly different antioxidant and free radical scavenging powers.

Regarding the independent or synergistic effects of MPE extract, and Curcum, Table 3 presents the ameliorative effects both of MPE extract and *Curcuma* on BMI as a measure of obesity. While MPE induces a remarkable decrease in BMI, *Curcuma* either alone or in combination was more effective This can find support in the work of Abuzaid et al. [38] in which a remarkable decrease of body adipose tissues was recorded in MPE due to the significant inhibition of fatty acid synthase in control obese fed high caloric diet. The remarkable decrease of body weight gain and BMI in *Curcuma*-treated either independently or in combination with MPE can find support in the work of Kim et al. [39] in which administration of Curcuma suppressed body weight increase and decreased white adipose tissue weight, serum triglyceride, and cholesterol in high-fat diet-induced obese rats. These results were concomitant with the reduction of adipocyte differentiation and lipogenesis through the downregulation of the mRNA expressions of fatty acid synthase, acetyl-CoA carboxylase, adipocyte protein 2, and lipoprotein lipase, together with the upregulation of β-oxidation enzymes.

Looking specifically at central obesity [40], higher levels of IL-5, IL-6, IL-12, IL-13, and IFN-γ were reported in participants with abdominal obesity compared to those without. In another study, IL-10 as an anti-inflammatory cytokine was significantly increased, whereas Tumor necrosis factors TNF-α and IL-6 decreased following calorie restriction [41]. Regarding the therapeutic effects of MPE and *Curcuma* either independently or synergistically, Figure 1 shows the highly significant anti-inflammatory effect of both extracts. The reported anti-inflammatory effects of MPE reported in the present study are in good agreement with the recent work of [42] in which they highlighted the anti-inflammatory, antioxidant, neuroprotective, and mitochondrial augmenting properties of MPE by considering pharmacotherapeutic studies on animal models. These effects can be explained on the basis that MPE is highly rich with many polyphenol-subclasses, such as xanthones and catechins [43]. Xanthones are reported to be higher in the pericarp compared to the palatable aril part of the fruit; the pericarp contains 10 folds with higher phenolic compounds and 20 folds higher antioxidant activity [44]. Xanthone is one of the important natural antioxidants; *α-mangostin* and *γ-mangostin* are the most prominent in MP. It has been studied in recent years and reported that it possesses anticancer, anti-inflammatory, antibacterial, and cardio-protective activities [45,46,47].

Taken together *Curcuma* with gallic acid, ascorbic acid, or xanthone, is assumed to get higher beneficial effects than taken independently. The synergistic effects of *Curcuma* and MPE as a source of xanthones were investigated in the current study. Figure 1 demonstrates the much higher anti-inflammatory effects of *Curcuma* and MPE in combination than each alone. This is not in good agreement with the work of Naksuriya and Okonogi [48] in which they concluded the avoidance of consuming *Curcuma* in combination with ascorbic acid, or xanthones. But the independent and synergistic effects of MPE can find great support in the work of Udani et al. [25] in which daily consumption of the mangosteen juice blend induces a significant reduction of C-reactive protein (CRP) level as a marker of chronic inflammation in humans. Gutierrez-Orozco and Failla emphasized the current progress in the anti-inflammatory research of mangosteen extracts, compounds, and products [49]. However, clinical trials focusing on the anti-inflammatory effect of mangosteen are limited.

Leptin as a hormone with a key role in food intake and body weight homeostasis is commonly elevated in obesity, a condition known as hyperleptinemia [50]. This was ascertained through our recently published work and other recent studies in which HFD-induced obesity in rats demonstrate a significantly higher leptin level and leptin resistance [51]. The use of drugs or specific bioactive food components with anti-inflammatory properties to reduce the inflammatory state associated with obesity, especially in the hypothalamus, may help to overcome leptin resistance. Although leptin is capable of controlling appetite and weight gain in humans and rodents, through the central circuits in the hypothalamus and suppressing food intake as well as speeding up energy expenditure, in case of overfeeding or an increase in calorie ingestion, the increased flux of glucose in the muscle and adipose tissues triggered peripheral insulin resistance and increase of leptin biosynthesis, but it is failed to reduce feeding behavior and prevent weight gain due to leptin resistance [52,53]

Atherogenesis is the process of forming plaques in the arteries. It is related to hypercholesterolemia, referring to elevated total and LDL blood cholesterol usually leading to coronary heart disease (CHD). Wihastuti et al. [54] reported that mangosteens and their derivatives have potent anti-atherogenic pharmacological activities. This is in good agreement with our obtained data in which MPE was more effective than *Curcuma* in ameliorating the significantly higher leptin in obese rats (Figure 2). Of course, this was reflected in the synergistic effect of MPE and *Curcuma* which show the highest anti-atherogenic effect compared to each alone [55].

Obesity in middle age has also been related to Alzheimer’s disease (AD) as one of the most prevalent neurological disorders. The relationship between impaired leptin signaling pathway and the onset of AD has been investigated with the blood-brain barrier (BBB) having a great and critical role. This usually occurs through the development of slow glutamate excitotoxicity in obese due to a surplus level of glutamate as the major excitotoxic neurotransmitter in the brain [56,57]. In our previous published work, a 79% increase in brain glutamate was reported in HFD-induced obese rats. Fritz et al. [58] observed that obesity was related to abnormal glutamate synaptic transmission as well as greatly enhanced dopamine transmission throughout the dorsal striatum of the brain. They proved that HFD consumption in mice resulted in focally sustained excitatory postsynaptic current (EPSC), due to the decreased glutamate buffering mechanism of glutamate through conversion to glutamine or gamma amino butyric acid (GABA), death of glutamate receptors NMDAR, due to the overstimulation with the surplus level of glutamate.

Figure 3 also presents the remarkable but non-significant decrease of whole-brain glutamate in the three treated obese groups. The reported decrease of glutamate in MPE -treated rats can find support in the study [59] which proves the effectiveness of MPE in reducing glutamate-induced neurotoxicity in a murine hippocampal neuronal line (HT22 cells) through a signaling pathway facilitated by its antioxidant effect. Additionally, it is in good agreement with the recent work of Do and Cho [60] in which different preparations of MPEs and MP diet were found to inhibit neurotoxicity and oxidative stress induced by amyloid β, NMDA glutamate receptors, H_2_O_2_ or other stimuli in in vitro and in vivo studies.

It is well known that dopamine and serotonin as neurotransmitters play important roles in homeostatic signaling [61]. Numerous studies, in rodents and humans [62,63]. have shown that experimental inhibition or stimulation of both transmitter signaling is associated with changes in feeding behavior, a stimulus to eat, reward learning, and energy expenditure. On the basis of these and other observations, it has been hypothesized that disturbed feeding behavior in obesity is caused by alterations in central dopamine and serotonin systems [64]. Additionally, Katy et al. [65] reported by using neuroimaging trials, the remarkable decrease of serotonin in obese compared to overweight and lean controls which supports the contribution of synaptopathy in the altered feeding behavior in obese. This is not in accordance with Abu-Taweel, the investigation [66] which demonstrates the ameliorating effect of Curcumin on 5 HT-depleted levels in mercury chloride-intoxicated rats.

While independent supplementation of MP extract was ineffective in ameliorating the significantly higher brain dopaminergic signaling in obese rats, both *Curcuma* alone or synergistically with MPE was potent in decreasing brain dopamine. This is in good agreement with multiple studies which prove the neuroprotective effects of *Curcuma* against Parkinson’s disease and other neurological disorders [67,68,69,70]. As *Curcuma* either alone or in combination was more effective in decreasing the BMI (BMI of 0.78 and 0.79 g/cm^2^ respectively) compared to control obese or even MPE-treated rats (BMI of 0.85 and 0.81 g/cm^2^). This might help to suggest that the anti-obesity effect of MPE might be through another mechanism which is apart from the dopaminergic signaling.

The inter-relationship between obesity, neuroinflammation, and brain neurotransmitters can be easily observed through the use of multiple regressions as a statistical tool. The change of IL-6 as a dependent variable was affected by IL-12, dopamine, and leptin as independent or predictor variables, and the change of leptin as a marker of obesity was greatly affected by both pro-inflammatory cytokines (IL-6 and IL-12, serotonin, and body weight (Table 4). This can find support in the recent work [47] in which they postulate that chronic low-grade inflammation, as detected in obesity, also distresses the brain, which may facilitate further weight gain.

The ROC analysis data of the three treated groups ascertained the usefulness of most of the measured variables as predictive markers of the therapeutic effects of mangosteen and *Curcuma* either independently or in combination. Almost all the recorded ROC-AUCs range between 0.8 and 1.0 with very high sensitivity and specificity (Table 5).

## 5. Conclusions

The evidence of the bioactivity and therapeutic effects of MP is rapidly developing. This fruit has produced promising results in HFD-obese animals and has been demonstrated to have anti-obesity, anti-inflammatory, and neuroprotective properties. Future research should encourage human clinical trials to discover the risks and benefits of using MP and to evaluate the potential for translation into clinical care.

## Figures and Tables

**Table 1:** Qualitative phytochemical analysis of the solvent fractions of the methanol extracts of *G. mangostana* pericarp and *C. longa* rhizomes.

Phyto-Constituents	*G. mangostana* Pericarp	*C. longa* Rhizomes
Phenolic compounds	+++	+++
Flavonoids	+++	+++
Xanthones	+++	+
Tannins	++	++
Phytosterols	++	++
Alkaloids	-	-
Saponins	-	-

+ = Present in mild amount, ++ = Present in moderate amount, +++ = Present in large amount, - = Absence.

**Table 2:** Estimation of total phenolic and flavonoid content and antioxidant activity of the methanol extracts of *G. mangostana* pericarp and *C. longa* rhizomes.

Sample	TPC (mg GAE/g Dried Extract)	TFC (mg CAT/g Dried Extract)	RP (Absorbance at 80 µg mL^−1^)	DPPH IC_50_ (µg mL^−1^)	OH IC_50_ (µg mL^−1^)
*G. mangostana*	86.23 ± 0.62 ^a^	137.24 ± 1.05 ^b^	2.123 ± 0.05 ^b^	10.35 ± 0.46 ^c^	12.37 ± 0.62 ^a^
*C. longa*	72.02 ± 0.22 ^b^	174.24 ± 0.20 ^a^	2.542 ± 0.03 ^a^	7.48 ± 0.52 ^b^	24.26 ± 0.25 ^c^
CAT	-	-	2.34 ± 0.06 ^c^	4.45 ± 0.10 ^a^	14.67 ± 0.25 ^b^

CAT, catechin; TCP, Total phenolic content; GAE, gallic acid; TFC, Total flavonoid content; RP, Reducing power; DPPH, 1,1-diphenyl-2-picrylhydrazyl radical scavenging; OH, Hydroxyl radical scavenging. Means in each column with different subscript letters (a–c) differ significantly (*p* < 0.05).

**Table 3:** Independent and synergistic effects of *C. longa* and *G. mangostana* extracts on BMI of lean and HFT-induced obese rats.

Parameters	Groups (n)	Mean ± SEM	Percentage Change	*p* Value ^a^
Initial BW (g)	Control obese (5)	458.60 ± 15.95	100.00	
	MPE-treated (5)	405.00 ± 7.46	−4.62	N.S
	*Curcuma*-treated (5)	411.20 ± 9.42	−0.83	N.S
	Synergistic-treated (5)	392.00 ± 17.27	−12.19	˂0.05
Final BW (g)	Control obese (5)	528.40 ± 15.17		
	MPE-treated (5)	504.00 ± 6.64		
	*Curcuma*-treated (5)	524.00 ± 9.68		
	Synergistic-treated (5)	464.00 ± 17.08		
Weight gain (g)	Control obese (5)	69.80 ± 2.33	100.00	
	MPE-treated (5)	99.00 ± 1.00	−11.69	˂0.05
	*Curcuma*-treated (5)	112.80 ± 1.39	−10.34	˂0.05
	Synergistic-treated (5)	72.00 ± 1.38	−14.52	˂0.05
BMI (g/cm^2^)	Control obese (5)	0.86 ± 0.09	100.00	
	MPE-treated (5)	0.81 ± 0.06	−5.49	N.S
	*Curcuma*-treated (5)	0.78 ± 0.03	−9.09	N.S
	Synergistic-treated (5)	0.79 ± 0.07	−7.46	N.S

^a^ Refer to the sig. difference between each group and control obese rats.

**Table 4:** Multiple Regression using the Stepwise method for the measured variables.

Dependent Variable	Predictor Variable	Coefficient	S.E.	*p* Value	Adjusted R^2^	95% CI	
						Lower	Upper
IL-6	IL12 (pg/mL)	0.895	0.274	0.002	0.974	0.340	1.450
	Dopamin (ng/mL)	0.340	0.140	0.020		0.056	0.624
	Leptin (ng/mL)	23.143	10.650	0.036		1.543	44.742
Leptin	IL12 (pg/mL)	0.016	0.003	0.000	0.982	0.009	0.022
	IL6 (pg/mL)	0.005	0.002	0.009		0.001	0.009
	Serotonin (ng/mL)	−0.007	0.002	0.005		−0.013	−0.002
	BW	0.003	0.001	0.029		0.000	0.005

**Table 5:** ROC-Curve of all parameters for Obese.

Parameters	Groups	AUC	Cut-Off Value	Sensitivity %	Specificity %	*p* Value	95% CI
Serotonin (ng/mL)	Control		55.000	100.0%	100.0%	0.009	1.000–1.000
	MPE-treated	1.000	53.500	100.0%	100.0%	0.009	1.000–1.000
	*Curcuma*-treated	1.000	51.500	100.0%	100.0%	0.009	1.000–1.000
	Synergistic-treated	0.840	51.000	80.0%	80.0%	0.076	0.580–1.100
Dopamin (ng/mL)	Control		60.500	100.0%	100.0%	0.009	1.000–1.000
	MPE-treated	1.000	67.000	100.0%	100.0%	0.009	1.000–1.000
	*Curcuma*-treated	0.960	53.000	100.0%	80.0%	0.016	0.843–1.077
	Synergistic-treated	0.700	60.000	100.0%	60.0%	0.296	0.337–1.063
Glutamate (ng/mL)	Control		49.500	100.0%	100.0%	0.009	1.000–1.000
	MPE-treated	0.800	47.165	80.0%	100.0%	0.117	0.449–1.151
	*Curcuma*-treated	0.800	49.000	80.0%	100.0%	0.117	0.449–1.151
	Synergistic-treated	0.840	44.000	80.0%	80.0%	0.076	0.580–1.100
IL12 (pg/mL)	Control		91.910	100.0%	100.0%	0.009	1.000–1.000
	MPE-treated	1.000	79.765	100.0%	100.0%	0.009	1.000–1.000
	*Curcuma*-treated	1.000	80.550	100.0%	100.0%	0.009	1.000–1.000
	Synergistic-treated	1.000	72.495	100.0%	100.0%	0.009	1.000–1.000
IL6 (pg/mL)	Control		180.995	100.0%	100.0%	0.009	1.000–1.000
	MPE-treated	1.000	145.125	100.0%	100.0%	0.009	1.000–1.000
	*Curcuma*-treated	1.000	147.960	100.0%	100.0%	0.009	1.000–1.000
	Synergistic-treated	1.000	134.970	100.0%	100.0%	0.009	1.000–1.000
Leptin (ng/mL)	Control		2.240	100.0%	100.0%	0.009	1.000–1.000
	MPE-treated	1.000	1.775	100.0%	100.0%	0.009	1.000–1.000
	*Curcuma*-treated	1.000	1.980	100.0%	100.0%	0.009	1.000–1.000
	Synergistic-treated	1.000	1.510	100.0%	100.0%	0.009	1.000–1.000
BMI	Control		0.705	100.0%	100.0%	0.009	1.000–1.000
	MPE-treated	1.000	0.695	100.0%	100.0%	0.009	1.000–1.000
	*Curcuma*-treated	1.000	0.755	100.0%	100.0%	0.009	1.000–1.000
	Synergistic-treated	1.000	0.660	100.0%	100.0%	0.009	1.000–1.000

## Data Availability

The datasets of the current study are available from the corresponding author on reasonable request.

## Abbreviations

Area under the curve, AUC; body mass index, BMI; high-fat diet, HFD; glutathione, interleukin, IL; Mangosteen pericarp, MP; Mangosteen pericarp extract, MPE; N-methyl-D-aspartate receptors, NMDA-R; phosphate-buffered saline, PBS; reactive oxygen species, ROS; receiver operating characteristics, ROC; World Health Organization, WHO.
